# Transient RNA–protein interactions in RNA folding

**DOI:** 10.1111/j.1742-4658.2011.08094.x

**Published:** 2011-05

**Authors:** Martina Doetsch, Renée Schroeder, Boris Fürtig

**Affiliations:** Department of Biochemistry and Molecular Cell Biology, Max F. Perutz Laboratories, University of ViennaAustria

**Keywords:** mode of binding, proteins that promote RNA folding, RNA chaperones, RNA folding problem, transient interactions

## Abstract

The RNA folding trajectory features numerous off-pathway folding traps, which represent conformations that are often equally as stable as the native functional ones. Therefore, the conversion between these off-pathway structures and the native correctly folded ones is the critical step in RNA folding. This process, referred to as RNA refolding, is slow, and is represented by a transition state that has a characteristic high free energy. Because this kinetically limiting process occurs *in vivo*, proteins (called RNA chaperones) have evolved that facilitate the (re)folding of RNA molecules. Here, we present an overview of how proteins interact with RNA molecules in order to achieve properly folded states. In this respect, the discrimination between static and transient interactions is crucial, as different proteins have evolved a multitude of mechanisms for RNA remodeling. For RNA chaperones that act in a sequence-unspecific manner and without the use of external sources of energy, such as ATP, transient RNA–protein interactions represent the basis of the mode of action. By presenting stretches of positively charged amino acids that are positioned in defined spatial configurations, RNA chaperones enable the RNA backbone, via transient electrostatic interactions, to sample a wider conformational space that opens the route for efficient refolding reactions.

## The RNA folding problem

RNA folding is the crucial process that connects RNA synthesis to RNA function. Many (non)coding RNAs and *cis*-acting elements within RNAs have to adopt complex three-dimensional structures to exert their roles within given cellular processes [1]. The structure–function relationship that highlights the importance of a defined RNA structure was first elaborated for tRNAs, for which several conformers coexist *in vitro*. Only one of these conformers (the biologically functional structure) can be aminoacylated and thus serve as a transfer molecule during translation [2], demonstrating the fact that only a single defined structure is able to perform the biological task. Recently, increased attention has been given to RNA molecules that adopt two functional forms – riboswitches and RNA thermometers. Both types of RNA molecule are able to sense environmental conditions within the cell and subsequently to adopt a certain structure that, in turn, leads to a functional response [3]. Riboswitches are structural elements of mRNAs that are sensitive to the concentration of a given metabolite modified by the protein translated from the mRNA itself. Via binding to an aptamer region (which is accompanied by induced structural rearrangements within the RNA), the metabolite can directly influence the regulation of the underlying gene. RNA thermometers are temperature-dependent secondary and tertiary structures formed by mRNAs that serve as on–off switches for mRNA translation. Here, different temperature-dependent structures of the same molecule exert opposite functions, namely either the blocking or presenting of binding sites for the ribosome [4]. These are just a few examples of the necessity for RNAs to precisely fold into defined structures, which are either the subject of or key components in RNA synthesis and maturation, translation, catalysis, and riboprotein complex formation. The folding of an RNA molecule into a specific structure is a slow process [2,5–7]. Because RNA is composed of only four nucleic acid building blocks, forming complementary pairs (A·U and G·C), and because, within RNA molecules, guanosine bases can pair with uridine bases without disrupting helical structures, a single RNA sequence can adopt many alternative secondary structures. This makes it difficult to define a unique fold, and leads to a rugged energy folding landscape [8–10]. The formation of entropically favorable local structures often leads to topological frustration; that is, the formation of various possible and stable but non-native secondary structural elements in the RNA often prevents the rapid establishment of tertiary interactions [7]. Therefore, RNAs are easily trapped in the form of transient intermediates, and these non-native structures slow down the folding process. As a consequence, RNA molecules pause at many kinetic traps on their folding pathway. This phenomenon has been referred to as the RNA folding problem [11]. RNA folding is most rapid when secondary and tertiary interactions within the RNA molecule are energetically balanced over the whole molecule. This can be achieved either by changes in the nucleotide sequence (introduction of mutations in experiments [12]) or by interactions with extrinsic factors [13,14].

Many factors influence the kinetics of RNA folding reactions. Environmental variables, such as temperature or the speed of synthesis and decay of the RNA molecule [15,16], are major determinants of the folding kinetics. Further factors that affect the speed and reaction route of RNA folding are ligands that interact with the RNA molecule. Such ligands can be metal ions [17], small molecules such as polyamines [18], and RNA-binding proteins [19,20].

The mechanisms by which proteins shape the RNA folding pathway can be subdivided into two main classes [19,21]. The first class is characterized by specific interactions between the protein and the RNA that lead to tight and stable functional complexes. This mechanism can be described either by a nucleation model or by a structure capture model. In the first model, the RNA folds around a given RNA binding platform provided by the protein cofactor. Conversely, the structure capture model assumes that, without the ligand, the RNA adopts many different transient interconverting conformations in dynamic equilibrium [22]. One conformation of the ensemble represents the RNA in the ligand-bound state. This specific conformation is recognized by the protein, interacts with it to form a stable complex, and is thereby removed from the conformational equilibrium [23].

The second mechanistic class of protein-assisted RNA folding is characterized by weak, nonspecific interactions. Here, the transient interaction of proteins with the RNA molecule destabilizes misfolded intermediates and lowers the free energy of transition states between conformations. As a consequence, a smoother energy landscape is produced that increases the rate of folding and the probability that a molecule will find its native structure. In this review, we will focus on those proteins that undergo transient interactions with RNA molecules during their folding process or during their assembly into RNP complexes.

## Static versus transient interactions

RNA folding reactions can be modulated either by tight binding to proteins, establishing a functionally static RNA·protein complex, or by transient interactions with proteins that dissociate from the RNA after a stable conformation is established. Generally, transient interactions are most important in reactions where a high turnover is required and the slow folding of one component is detrimental to the assembly of a higher RNP complex (e.g. spliceosome or ribosome). The folding-assisting protein has to dissociate to enable the RNA to function when it has adopted its functional conformation [24].

To best describe the nature of transient interactions, they are compared with static interactions, as they have an exactly opposite character. Tight complexes have long lifetimes (seconds or longer), whereas RNA-protein complexes based on transient interactions have lifetimes ranging from microseconds to milliseconds. Typically, the characteristic affinities for two binding partners that only interact transiently are found to be in the micromolar to millimolar range, because the off-rates are high (*k*_off_ ≥ 0.2 s^−1^) [25]. A further way of describing macromolecular complexes is by the molecular interface of the interacting molecules. In common stable complexes between RNAs and their specific RNA-binding proteins, such as the RRM domains [26], KH domains [27], CCHH-zinc fingers [28], dsRBDs [29], and PAZ domains [30], the interfaces are tightly packed and provide perfect complementarity between the binding partners. In contrast, interfaces of transient complexes are often not densely packed, and water can more easily gain access to the RNA–protein interface to increase the dissociation process. The promiscuity often reported for proteins that interact only transiently with RNA is achieved by the lack of geometrically complementary interfaces. Charged residues are frequently found in both static and transient complex interfaces, but in transient interfaces they are more often located at the perimeter. The presence of lysines and arginines to oppose the negatively charged sugar-phosphate RNA backbone is important, and they are found 1.5 and 1.4 times more often than in interfaces of protein-protein complexes [31]. Nonetheless, an exact match in transient complexes is not assumed, as it would prevent the disintegration of the complex.

## Proteins help RNAs to fold and unfold

As mentioned above, optimal folding rates of RNA require an energetic balance between local and global interactions within the molecule [7]. If this balance is not intrinsic to the molecule itself, it can be achieved by the interaction of the RNA with proteins. If the Δ*G*^local^/Δ*G*^global^ ratio is far from unity and thereby unbalanced (meaning that the formation of local structures is more favorable than global interactions – assuming that both values have negative signs), then two possible scenarios of how proteins may contribute to the successful achievement of a Δ*G*^local^/Δ*G*^global^ ratio close to unity can be envisioned – either the protein stabilizes structure elements that are responsible for the formation of the global structure of the RNA (such as tertiary interactions) by recognition and subsequent binding to them, or the protein destabilizes local interactions (which mainly involve secondary structure elements), e.g. by opening base pair interactions.

Within the framework of this theoretical consideration, three types of proteins have been found to promote RNA folding: (a) specifically binding proteins, which recognize and bind certain RNAs and thus stabilize the RNA structure, thereby forming a stable RNA-protein complex; (b) proteins with RNA chaperone and annealing activity, which interact only transiently with RNAs without the recognition of a specific structure or sequence, thereby promoting folding via unfolding or via annealing acceleration; and (c) RNA helicases, which accelerate the unwinding of many RNAs under conditions of ATP binding and hydrolysis.

Here, we summarize the properties of the three protein classes, with the main focus being on RNA chaperones and annealer proteins.

### Specifically binding proteins

A specific protein cofactor binds to its RNA target through well-defined structural features, thereby stabilizing its native structure. Two scenarios have been shown or postulated – either the protein can bind to the RNA molecule when it has already adopted its correct structure, or the specific binder can interact with the RNA during its folding process and can accelerate folding or even nucleate the folding event. In a distinct mechanism, the protein may capture one specific conformation out of an ensemble of possible structures [22].

While the functional fold of the RNA molecule has not yet been achieved, the protein can interact transiently with the native RNA substrate. During this first encounter, the protein can perform unfolding activities reminiscent of RNA chaperone activities to support the folding process and to achieve specific binding. Furthermore, specific binders have been shown to exert RNA chaperone activity when encountering RNAs that do not contain the canonical binding motif. A well-studied example is the CBP2 protein from yeast mitochondria, which binds specifically to the bI5 group I intron [32]. The interaction of CBP2 with the intron RNA was studied with fluorescence resonance energy transfer, monitoring the dynamics of the RNA at a single-molecule level [33]. According to these studies, CBP2 stabilizes the native conformation, but additional, nonspecific interactions cause large conformational fluctuations in the RNA. Another example is the mitochondrial tyrosyl-tRNA synthetase Cyt-18 from *Neurospora crassa*, which binds specifically to group I introns, thereby stabilizing the three-dimensional structure of the RNA. The protein can display RNA chaperone activity when interacting with nonspecific RNAs [34,35]. In a fluorescence resonance energy transfer-based assay, Cyt-18 efficiently promoted strand displacement of an artificial 21mer RNA duplex [36].

### RNA helicases

DEAD-box proteins are RNA helicases that are ubiquitous in all RNA-mediated processes. They use ATP hydrolysis to (mostly sequence-independently) promote conformational changes in RNA molecules, to disrupt RNA structures in a nonprocessive way, and to accelerate structural transitions in RNAs and RNP complexes [37]. DEAD-box proteins also disrupt RNA–protein interactions [38,39], and some have been shown to promote duplex formation [40,41], which stresses their resemblance to proteins with RNA-annealing activity. DEAD-box proteins should therefore be considered as major players in RNA folding and in the assembly and functioning of RNP machines, mostly through transient interactions with the RNA.

DEAD-box proteins have low processivity when unwinding helices shorter than 25–40 base pairs [40], probably because their unwinding mechanism does not involve translocation, and nor does the ATP hydrolysis correlate with unwinding. High-resolution X-ray structures have given insights into the mechanism(s) of DEAD-box helicases. The binding sites for double-stranded RNA and ATP overlap, resulting in coupled binding of both molecules. Simultaneous binding forces the RNA into a bent conformation that is incompatible with duplex formation, suggesting that the induction of this bent state might be the initial step in strand separation by DEAD-box helicases [42,43]. Following this local duplex disruption, the bound ATP is hydrolyzed. Prior to ATP hydrolysis, single-stranded RNA is bound tightly to the protein. However, after ATP hydrolysis, conformational changes drive a cycle of regulated single-stranded RNA binding affinity transitions, so that protein and RNA dissociate [44].

### RNA chaperones and annealers

RNA annealer proteins are able to accelerate annealing of complementary nucleic acid sequences. RNA chaperones have the ability to destabilize formed RNA structures, which is measurable in strand displacement assays, and may additionally accelerate annealing. The hypothesis that RNA chaperones and annealers interact with their targets in a transient way is founded on four main observations, as follows. (a) By definition, sequence-nonspecific activity is inherent to RNA chaperones [11,45]. Although, for some RNA chaperones, specific substrates or preferred nucleotide compositions have been identified, these proteins can accelerate annealing or catalyze strand displacement for a large variety of nucleic acid sequences. Interactions with both DNA and RNA have been demonstrated for a number of RNA chaperones, such as nucleolin [46,47], hepatitis delta antigen [48,49], and NCp7 [50], and may apply to all proteins of this class. (b) The dissociation constants measured for RNA chaperones and the nucleic acid substrates used are mostly in the low micromolar range, and thus outside the range of specific interactions [51]. (c) Although RNA chaperones and RNA annealers do not share common motifs, they harbor domains or surfaces with many basic amino acids [48,50,52–56]. Both this feature and the often reported dependence of the activity on the ionic strength of the solution [50,57–59] hint at the interaction between the proteins' basic amino acids and the nucleic acid backbone via ionic forces. In fact, transient interactions are characterized mainly by long-range electrostatic interactions [60]. (d) For the human mRNA-binding protein hnRNP A1 [61], the *Xenopus laevis* protein X1rbpa [54], the trypanosome guideRNA-binding protein RBP16 [62], and the *Escherichia coli* protein StpA [63], an inverse or missing correlation between substrate binding strength and activity has been found. On the basis of the four above-mentioned observations, we hypothesize that the transient nature of RNA chaperone–RNA interactions is not a coincidence, but is in fact a prerequisite for the chaperone and annealing activity, and that it is the key to understanding the mechanism of protein-facilitated RNA folding. To develop this idea further, we concentrate on two proteins that have been studied in detail in this respect.

#### The HIV-1 transactivator of transcription (Tat) peptide is a potent nucleic acid annealer

The peptide Tat(44–61) is an 18-residue fragment of the HIV-1 Tat protein. Its sequence-nonspecific annealing activity was first described by Kuciak *et al.* (2008) [64]. Because of its basicity and its short length, we selected it as a model RNA annealer protein to study the mechanism of acceleration of annealing [65]. We found that Tat(44–61) efficiently annealed both short RNA and DNA substrates of different length and sequence. The annealing activity of the peptide was strongly inhibited at MgCl_2_ concentrations above 2 mm and at NaCl concentrations above 60 mm. Supporting the assumption of ionic interactions between peptide and RNA, the overall charge of the peptide was crucial for the activity, as the replacement of single basic amino acids with alanine resulted in the annealing rate constant decreasing by a factor of 2.3–3 as compared with the wild-type peptide. Thermodynamic calculations regarding the transition state of the reaction explained the importance of the overall charge for the activity – the total peptide charge determines the magnitude of peptide–RNA binding, owing to counterion release from the RNA backbone [66]. The resulting entropy increase of the system drives binding of the peptide to the RNA (and thus, indirectly, the acceleration of annealing). However, the extent of decrease of annealing acceleration caused by the single amino acid mutant peptides was not reflected in the dissociation constants as determined by filter binding. Besides the overall charge, we found an exact spatial arrangement of basic amino acids to be important for the activity – scrambled peptides with the same amino acid composition as the wild-type peptide showed decreased performance in our annealing assay. ^1^D^1^H-NMR spectra of a single-stranded RNA showed that, depending on the amount of peptide added, the Tat peptide induced a change in the population of coexisting and interchanging RNA conformations. The lack of intermolecular NOE connectivities indicated a short residence time of the peptide in the RNA-peptide complex, confirming the transient interaction between the molecules [65]. Taking all these results into account, we suggest that the Tat peptide, by interacting transiently with the RNA phosphates, alters the structure of the RNA substrate. It thus increases the probability of successful procession from the encounter complex of two RNA molecules to the transition state with the first-formed base pairs and consequently to the final RNA duplex. Whether the annealing activity of the Tat protein plays a role *in vivo*, such as transcriptional activation of the viral genome, remains to be elucidated.

#### The *E. coli* protein and RNA chaperone StpA

The nucleoid-associated protein StpA in the form of a heterodimer with its homolog H-NS shapes the structure and organization of the *E. coli* genome and thus regulates various genes [67]. Besides its association with DNA, StpA has been found to interact with many different RNA molecules without exerting any sequence specificity. Accordingly, a genomic SELEX failed to identify a specific substrate for StpA [63]. Moreover, StpA was identified as a protein displaying RNA chaperone activity. It is able to promote the proper folding of ribozyme molecules both *in vitro* and *in vivo*. Restricted proteolysis experiments demonstrated a modular architecture of the protein, with two separate structural and functional domains. Most data map the RNA interaction function to the C-terminal domain (CTD) of StpA. Accordingly, this domain alone is able to catalyze RNA folding, as demonstrated in various different assays. In order to exert RNA chaperone activity, both the full-length protein and the CTD must be present in concentrations close to the respective dissociation constants, which are usually in the micromolar range [68–70]. This means that, in assays, StpA is usually applied in molar excess over the RNA substrates, and that the RNA is most probably coated with several protein molecules, as opposed to a 1 : 1 stoichiometry. In contrast to the entropy transfer model, the CTD of StpA is a structured domain comprising two antiparallel β-strands and two terminal α-helices (B. Fürtig, unpublished results). The domain displays a highly positively charged surface. It can be shown that the interaction with the RNA takes place at the positively charged patches of the surface. Furthermore, those regions also represent the flexible residues within the protein domain. NMR data provide evidence that the interaction site on the RNA is the phosphate backbone. This is also in accordance with the demonstrated inhibitory effect of monovalent and divalent cations on RNA binding and RNA chaperone activity [63]. Interestingly, the interaction between the CTD and RNA can be monitored by solution-state NMR spectroscopy but not by classical electrophoretic mobility shift assay, even at very low salt concentrations. As the latter assay would require the formation of a stable complex, the formation of only transiently populated RNA·protein complex states can be inferred. Furthermore, the results of the NMR titration series also show that the interaction takes place in the fast-exchange regime, meaning that the *k*_off_ must be high (B. Fürtig, unpublished results). Interestingly, the StpA G126V mutant shows a dramatically reduced binding affinity, despite being more active in a chaperone assay than the wild-type protein [63]. Stressing the notion of transient interactions between StpA and RNA even further is the fact that the protein is dispensable after the refolding of an RNA molecule has occurred, and can be digested by proteinase K [69]. In all, these results lead to the conclusion that the transient nature of the interaction between RNA and protein is a prerequisite for the mode of action of (these) RNA chaperone(s).

As StpA and also its CTD alone can promote annealing as well as displacement of complementary RNAs, the question of which changes in the RNA are introduced during the transient interaction arises. Initial results indicate that the protein acts as an electrostatic lubricant that shields repulsive interactions within the RNA molecule. The protein thereby smooths the folding energy landscape. The direction of the RNA folding reaction (either annealing or displacement) is then no longer kinetically controlled, but instead follows the reaction route determined by thermodynamics.

#### A general annealing and chaperoning model

**Fig. 1:**
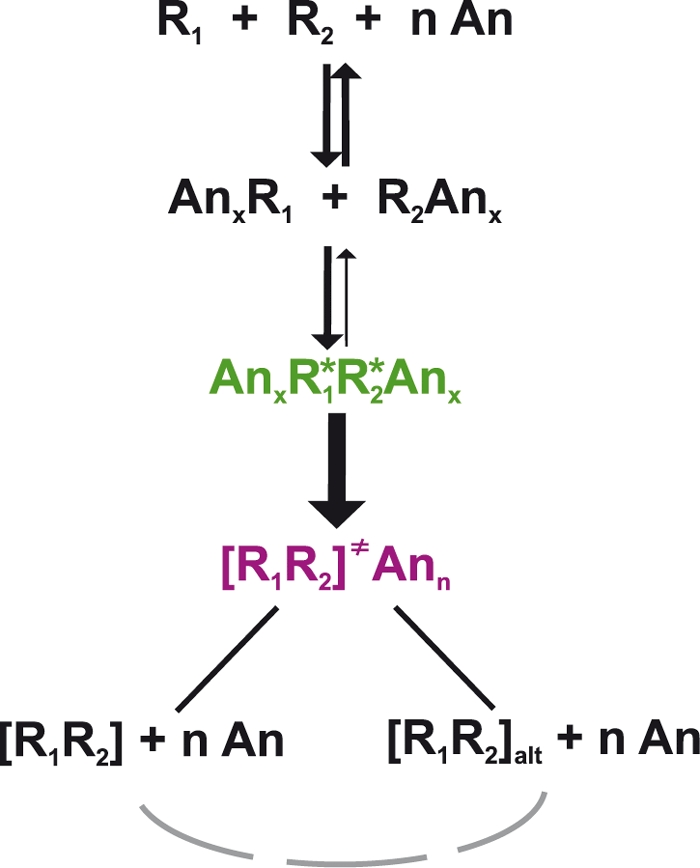
A generalized model for proteins that accelerate annealing and proteins capable of strand displacement (RNA chaperones). (A) In the RNA-only scenario, two complementary RNAs (R_1_ and R_2_) form an encounter complex and (once the necessary activation energy is reached and molecules show a favorable conformation and orientation) proceed to a transition state before they establish the RNA duplex. Apart from the thermodynamically favored duplex, alternative double strands (alt) can form. (B) Each RNA molecule is coated by several molecules of an annealer protein (An). The annealer protein supports the reaction by altering the structure of RNA molecules, which leads to annealing-competent RNA conformations. Thus, the fraction of encounter complexes that fall apart is decreased, and more encounters lead to successful procession to the transition state and, finally, the double strand. If the annealer protein has also strand displacement (SD) activity, it can reopen alternative structures, so that, eventually, only the thermodynamically favored duplex is found. (C) RNA duplexes that exceed a certain minimum stability will not disintegrate spontaneously. However, proteins with strand displacement activity destabilize such double strands by partially opening the duplex ends (indicated by parentheses). This allows an invading RNA, R_3_, to compete with R_2_ for base pairing with R_1_.

From the observations described above, we have delineated a general model for the mechanism of protein-facilitated annealing and strand displacement (Fig. 1).

To illustrate the mechanism of RNA annealing acceleration, we first consider the annealing of RNA in the absence of any supporting protein (Fig. 1A). Like other molecules that react with or bind to each other, RNA molecules form a transient encounter complex upon their first collision. According to the Arrhenius theory, the complex proceeds into a transition state only when the prerequisites of availability of the reaction activation energy, an appropriate RNA conformation and a suitable orientation of the molecules towards each other are fulfilled. Whereas the procession from the transition state into the final duplex is assumed to be very fast [71,72], the formation of the transition state can be – because of its high free energy – the rate-limiting step in nucleic acid annealing. We assume that this high free energy results from RNA conformational changes that have to occur prior to the formation of adjacent base pairs. In the presence of a protein with annealing activity, RNA molecules are ‘coated’ with this protein, owing to electrostatic attraction (Fig. 1B). The annealer protein, via transient interactions, alters the RNA structure in such a way that the probability of procession from encounter to transition state is increased. The result is an increase in the overall reaction velocity.

The strand displacement event resulting in an RNA duplex caused by a third, invading RNA molecule is often closely connected with the process of RNA annealing [73,74]. RNA chaperones destabilize double strands, starting from the ends or bulges of the base-paired region, and independently of the thermodynamic stability of the double strand (Fig. 1C). A third strand can utilize such destabilized regions as starting points for invasion. The concerted process of opening of the initial double strand and the annealing of the new duplex finally results in either the replacement of the original strand or the expulsion of the invading strand, according to the kinetics and thermodynamic situation. If the RNA chaperone also has annealing activity, it can catalyze the strand displacement event in two ways: by destabilizing edges and bulges, and by favoring the annealing reaction of the invading strand.

A clear advantage of transient interactions between RNA annealers/chaperones and their substrates is the low energy consumption of the reaction, especially in comparison with helicases, which have an ATP-dependent activity. Further advantages of transient interactions are a broad spectrum of substrates and the rapid availability of the protein for subsequent reactions. In order to avoid the general impairment of important cellular RNA structures, stringent regulation of expression and activity of these proteins is necessary. Thus, general RNA annealers and chaperones may be useful additions to the arsenal of specific RNA binders and helicases for the structural remodeling of RNA molecules.

## Abbreviations

CTD: C-terminal domain
Tat: transactivator of transcription
